# Fruit and Vegetable Consumption among Special School Students with Mild Intellectual Disability in Hong Kong

**DOI:** 10.5539/gjhs.v8n8p31

**Published:** 2015-12-17

**Authors:** W. K. Mok, T. K. Ling

**Affiliations:** 1 Department of Applied Science, Hong Kong Institute of Vocational Education, Vocational Training Council, Hong Kong, China

**Keywords:** fruit & vegetable consumption, students, mild intellectual disability, theory of planned behaviour

## Abstract

**Objective:**

The aim of this study was to predict the fruit and vegetable consumption intention of students with mild intellectual disability in Hong Kong by the application of Ajzen’s Theory of Planned Behaviour.

**Methods:**

50 students with mild intellectual disability (30 male and 20 female), ranging in age from 15 to 38 years, were participated in this study. By means of face-to-face interviews, demographic data, Food Preference and variables of Theory of Planned Behaviour, such as Attitude, Subjective Norm and Perceived Behavioural Control were measured.

**Results:**

20%, 28% and 10% students with mild intellectual disability were rated to be overweight, obese and severely obese respectively. The rest of 10% were classified to be underweight. Regarding the daily intake of fruit and vegetable, 96% students with mild intellectual disability failed to consume sufficient amount. The variables of Theory of Planned Behaviour explained 47.7% of fruit and vegetable consumption intention with significant factors of Attitude, Subjective Norm and Perceived Behavioural Control. Food Preference was found to be a useful construct and further improve the prediction by about 7% after incorporating into the model.

**Conclusions:**

Results of this study indicated that Theory of Planned Behaviour is a useful model to predict dietary intention of students with mild intellectual disability in Hong Kong. Food Preference was a significant predictor to model the intention of fruit and vegetable consumption among students other than Attitude, Subjective Norm and Perceived Behavioural Control.

## 1. Introduction

### 1.1 Intellectual Disability

Intellectual disability (ID) is a condition of incomplete development of the mind (World Health Organization (WHO), 1996). Schalock defined ID as a disability with significant limitations both in intellectual functioning and adaptive behaviour as expressed in conceptual, social and practical adaptive skills (Schalock et al., 2010). The impaired skills of cognitive, communication, motor and social abilities may lead to a reduction of ability to adapt the needs of daily living. The below average intellectual functioning limits the ability to take care of oneself, to make decisions and to be aware of personal health and safety (Rimmer & Yamaki, 2006). Referring to Table 1, ID is usually classified into 4 types (American Psychiatric Association, 2000), which are “mild”, “moderate”, “serve” and “profound” regarding the intelligence quotient level. According to the studies of Special Olympic, there were more than 200 million people with ID around the world (Special Olympic, 2009). The total number of people with ID in Hong Kong was estimated to be in the region of 71000 to 101000 out of a total population of 7.1 million, which represented a prevalence rate of about 1% to 1.4% (Census and Statistics Department of Hong Kong, 2015).

**Table 1:** The severity levels of ID with respected to the IQ scores and characteristics (WHO, 1996)

Levels	IQ Score	Characteristics
Mild ID	50-69	Able to use speech for everyday purposes May learn to read and write basically Difficult in academic learning Independent in self-care and practical skills Behavioural, emotional and social difficulties
Moderate ID	35-49	Limited in the use of language Simple communication Difficult in handling money Retarded in self-care and motor skills Able to perform simple practical work
Severe ID	20-34	Similar to moderate ID A degree of motor impairment Little or no communication
Profound ID	Below 20	Do not able to comply with instructions Immobile and incontinent Close supervision required
Other ID	/	Impossible to assess the degree of ID
Unspecified ID	/	With evidence of ID but insufficient information to assign into one of categories

### 1.2 Obesity among People with Intellectual Disability

It is well known that obesity increases the risk of cardiovascular diseases and also relates to type II diabetes, stroke, certain cancers and some other chronic health problems (WHO, 2000). The study by Yamaki revealed that 34.6% of people with ID were obese and this value was much higher than the figure 20.6% of general population within 1997 to 2000 in United Status (Yamaki & Steven, 2005). Heller also found that people with ID tended to have a higher rate of obesity and poorer nutrition, consuming high-fat foods but also inadequate in fruit and vegetable in general (Heller, McCubbin, Drum, & Peterson, 2011). Some studies concluded that poor eating pattern was one of the vital causes of obesity (Rimmer & Yamaki, 2006). Besides, Chan reported that people with ID were usually with a higher score of body mass index (BMI) than normal group of people in Hong Kong (Chan, 2014). Several studies revealed that people with ID would have a higher rate of heart diseases, diabetes and cancer than the general population (Wong, 2011; Morin, Merineau-Cote, Ouellettee-Kuntz, Tasse, & Kerr, 2012). People with ID were also associated with a shorter lifespan and higher mortality rate (Daily, Ardinger, & Holmes, 2000).

### 1.3 Fruit and Vegetable Consumption

Fruit and vegetable (F&V) offered a wide range of health-promoting nutrients as they are excellent sources of carotenoids and enriched in vitamin C, folate, potassium and phytochemicals. Intake of adequate F&V daily is associated with a reduced risk of cardiovascular diseases, including heart attack and stroke. Some of them may be protective against certain types of cancer because of the prevention of oxidative damage from free radicals to cellular components by available antioxidants (Wise, 2001). Dietary fiber in F&V helps to reduce the risk of type-II diabetes and obesity as it promotes healthy lipid profiles and glucose tolerance, which are essential to prevent most health problems (Central Health Education Unit, 2012). According to Department of Health, the amount of adequate daily F&V intake per person is set to be “2 servings of fruit plus 3 servings of vegetable” in Hong Kong. Throughout this research, this criteria was taken as the passing amount of sufficient daily F&V intake per person.

Low F&V consumption was identified as one of the risk factors for negative health consequences such as cardiovascular diseases and cancers which were the leading causes of deaths in Hong Kong. According to the behavioural risk factors survey conducted by Centre for Health Protection of Hong Kong in 2014, around 81% of respondents did not consume adequate F&V (Centre for Health Protection). The report further revealed that 47.7% and 30.5% of respondents consumed even less than one fruit and one bowl of vegetable a day respectively. Female was likely to eat more F&V than male. Some studies in United States indicated that only about 6% of people with ID consumed enough F&V (Draheim, Stanish, Williams, & McCubbin, 2007; Heller, McCubbin, Drum, & Peterson, 2011). However, the F&V consumption behaviour among people with mild ID in Hong Kong is not clearly studied.

### 1.4 Theory of Planned Behaviour and Fruit & Vegetable Consumption

Fishbein and Cappella suggested that application of behavioural theories could help to generate effective health intervention programmes and design persuasive communication (Fishbein & Cappella, 2006). Theory of Planned Behaviour (TPB) is designed to predict human behaviour (Ajzen, 2011). It is one of the models which is widely adopted in predicting human behaviour (Brickell, Chatzisarantis, & Pretty, 2006; Mok & Lee, 2013). Various works have applied TPB to study F&V consumption behaviour and supported suitability of the theory (Bogers, Brug, Van Assema, & Dagnelie, 2004; Kothe, Mullan, & Butow, 2012; Mok & Man, 2013). Hagger pointed out that few significant cross-culture differences in the structural relations among the TPB constructs were spotted (Hagger et al., 2007). Although TPB model has been proved having high validity to predict F & V consumption, limited research has been done on the people with mild ID.

**Figure 1:**
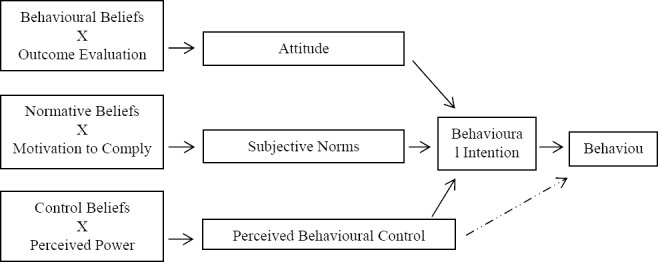
Theory of planned behaviour (TPB)

TPB is a model generated from the Theory of Reasoned Action (TRA) (Ajzen, 2011), which assumes that the specific behaviour is determined by intention to perform it (Fishbein & Ajzen, 2010). The model extended the TRA by supplementing the Perceived Behavioural Control (PBC) because TRA experiences difficulty in explaining behaviours in which a person does not have volitional control over it. Armitage and Conner made an excellent review on TPB (Armitage & Conner, 2001). Basically, there are three antecedents of Behavioural Intention, which are Attitude, Subjective Norm and PBC as shown in Figure 1.

Behavioural Intention is the measure of an individual’s willingness to perform a desired behaviour. Based on the rationale of TPB model, Behavioural Intention is a proximal predictor of behaviour (Ajzen, 2011). The higher Behavioural Intention towards a specific behaviour, the more likely is that the person engaged in that behaviour. Attitude is the measures of the salient behavioural beliefs. It is the function of an individual who perceived the likelihood of outcome due to performing a behaviour and individual’s evaluation of that outcome (Conner & Sparks, 2005). Fishbein and Ajzen suggested that Attitude could be classified into affective (eg. unpleasant/pleasant etc.) and instrumental elements (eg. harmful/beneficial) (Fishbein & Ajzen, 2010). Subjective Norm measures the effect of social influence exerted on individuals. It is the perception that individuals perceived the pressure whether they perform or not a specific behaviour (Ajzen, 2011). Some studies suggested that Subjective Norm was a rather weak predictor in adults. However, teenagers were more subject to significant others such as peer and parents (Godin & Shephard, 1986). Perceived Behavioural Control is the measure of ‘*people’s perception of the ease and difficulty of performing the behaviour of interest*’ (Ajzen, 2011). The extent that individuals could successfully transform their behavioural intention into actual behaviour greatly depends on their volitional control over that behaviour. Because of the higher control on behaviour, the intention on preforming behaviour would be higher (Fishbein & Ajzen, 2010). Measures of Perceived Behavioural Control reflected an individual’s control over a specific behaviour.

**Figure 2:**
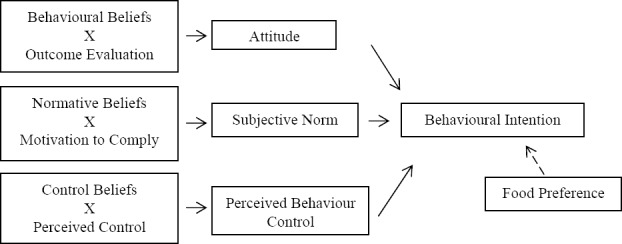
Theory of planned behaviour (TPB) with extension

Perceptions about taste had been found to be one of the psychosocial factors of adequate F&V consumption for both children and adults but omitted in Ajzen’s TPB model (Reinaerts, De Nooijer, Candel, & De Vries, 2007; Hartman, Wadsworth, Penny, Van Assema, & Page, 2013). Some studies also pointed out this possible relationship (Krebs-Smith et al., 1995; Van Duyn et al., 2001; Blanchette & Brug, 2005; Larson, Laska, Story, & Neumark-Sztainer, 2012) In order to study the association between F&V consumption and food perceptions, the perception of “tastiness of F&V” was measured and named as Food Preference to be an additional predictor in this study. The TPB with additional construct, Food Preference, is shown in Figure 2.

Students with ID were vulnerable for health disparities but they were usually having less attention and resources in the community. Lack of studies focusing on the health and dietary behaviours of minority group is not surprising. The aim of the present study was to apply the TBP model with extension to predict F&V consumption intention amongst special school students with mild ID in Hong Kong.

## 2. Materials and Methods

### 2.1 Participants

The current research was carried out during the 2012-2013 academic year. One government-funded special school, which provides a wide range of industry-specific skills training and rehabilitation for students with mild ID in Hong Kong, was selected to participate in this case study. With response rate of 67.5%, there were a total of 50 (30 male and 20 female) students enrolled out of 86 invitations, ranging in age from 15 to 38 years. All of the participants were Chinese with mild ID. Data was taken from the corresponding students by means of face-to-face interviews. Training session was given to all interviewers by professional social worker on how to deal with students with mild ID.

### 2.2 Procedures

All subjects participated voluntarily together with parents’ consents. Ethical requirements for the collection of personal data of the Institute of Vocational Education were strictly followed.

Face-to-face interviews were conducted in January 2013 during normal school days. Two interviewers (one male and one female) handled one student only in every interview session. Height and weight of the participants were measured. Questionnaire of basic demographic information and TPB variables were asked in Cantonese with the aids of pictures and food models. Each interview session lasted for about half an hour.

### 2.3 Instruments

Questionnaire, consisting of three parts, was employed as a mean to measure students’ health behaviour. The basic demographic information included gender, height and weight was measured in the first part. In the second part and third part, TPB variables and Food Preference were measured accordingly.

Regarding the TPB variables, all items were measured by using 7 point Likert Scale. The questionnaire items were adapted from a measure used previously by Mok (Mok & Man, 2013). Pilot test was performed before the start of interviews, the overall internal reliability was within acceptable range, which the Cronbach’s α was.833.

### 2.4 Attitude

Behavioural Beliefs and Outcome Evaluation were indirect measures of Attitude. Examples of questions are ‘I believe that people acquired “2 servings of fruit plus 3 servings of vegetable daily” can __________.’ and ‘I believe that it helps me to __________ if I consumed “2 servings of fruit plus 3 servings of vegetable daily”.’ The scores given described as the level of differences. The Cronbach’s α for this section was .728.

### 2.5 Subjective Norm

Normative Beliefs and Motivation to Comply contributed participants’ Subjective Norm. Examples of questions are ‘regarding consumption of “2 servings of fruit plus 3 servings of vegetable” daily, my parent/family members (teachers/classmates or friends) are __________.’ There were two questions asked to measure participants’ subjective norm directly which were in the format of ‘I think most of people who are important to me would agree/support with me if I decided to consume “2 servings of fruit plus 3 servings of vegetable” daily.’ The internal reliability was satisfactory, Cronbach’s α was .851.

### 2.6 Perceived Behavioural Control

Control Beliefs and Perceived Power measured Perceived Behavioural Control indirectly. Examples of questions are ‘I think the below factors towards fruit and vegetable would be obstructed me to consume “2 servings of fruit plus 3 servings of vegetable” daily.’ and ‘I think it would not be obstructed me even below barriers towards fruit and vegetable occurred if I decided to consume “2 servings of fruit plus 3 servings of vegetable” daily.’ respectively. The internal reliability for this section was barely acceptable, Cronbach’s α was .519.

### 2.7 Behavioural Intention

Three questions were asked to measure behavioural intention. Questions are ‘I plan/try/decide to consume “2 servings of fruit plus 3 servings of vegetable” daily in the following month.’ The internal reliability, Cronbach’s α, was .676.

### 2.8 Extended Food Preference Model

‘I think fruit/vegetable is tasty.’, ‘I am likely to eat fruit/vegetable.’ and ‘I would consume fruit and vegetable rather than meats including seafood if I have a chance to select.’ were the questions asked for investigation of their food references. The internal reliability for this section was satisfactory, Cronbach’s α was .749.

## 3. Result

### 3.1 Demographics

There were 50 participants in total while 30 are male and 20 are female. 20%, 76% and 4% of them aged 15-18, 19-28 and 29-38 respectively (Age: M = 22.1, SD = 3.45). Regarding body mass index (BMI), the overall results were shown in Table 2 (BMI: M = 24.62, SD = 4.60). Only 32% of participants were in normal category of BMI. 10% of participants were reported to be underweight. Worst still, 20%, 28% and 10% of participants were found to be overweight, obese and even severely obese respectively.

**Table 2:** Distribution of BMI

Body Mass Index	Count	Percentage	Cumulative Percentage
Underweight (below 18.5)	5	10%	10%
Normal (18.5-22.9)	16	32%	42%
Overweight (23-24.9)	10	20%	62%
Obese (25-29.9)	14	28%	90%
Severely obese (30 or above)	5	10%	100%
Total	50	100%	

### 3.2 Fruit & Vegetable Consumption

Regarding fruit daily consumption, there were about one-third of participants consuming two or more servings (Daily fruit intake: M = 1.51, SD = 1.095). However, only 10% of participants have three or more servings of vegetable per day (Daily vegetable intake: M = 1.5 SD = .89). With reference to the recommendation of Department of Health, 96% of participants did not consume enough F&V daily. In addition, 22% and 12% of participants consumed less than one serving of fruit and vegetable daily respectively. Those data showed that students with mild ID had inadequate F&V consumption to attain health.

### 3.3 Gender Effect on Theory of Planned Behaviour Scores and Fruit & Vegetable Consumption

According to Table 3, it was found that male had a higher subjective norm score than female by independent *t*-test (Male: M = 12.033, SD = 1.721; Female: M = 10.958, SD = 2.04; p =.05). No significant difference was spotted in other constructs of TPB between male and female.

**Table 3:** Mean difference between gender and subjective norm

	Male Mean (SD)	Female Mean (SD)	P-value
Subjective Norm	12.033 (1.721)	10.958 (2.04)	.05 *

* p =.05

### 3.4 Descriptive Statistics of Theory of Planned Behaviour

Descriptive statistics and bivariate correlations for the variables of interest are shown in Table 4.

**Table 4:** Descriptive statistics for TPB with Inter-correlation between measures (n=50)

	BI	A	SN	PBC	FP	Mean	SD
Behavioural Intention (BI)	1	.613**	.561**	.511**	.617**	5.44	1.339
Attitude (A)	/	1	.619**	.505**	.610**	12.137	1.440
Subjective Norm (SN)	/	/	1	.335*	.372**	11.603	1.911
Perceived Behavioural Control (PBC)	/	/	/	1	.489**	9.751	1.647
Food Preference (FP)	/	/	/	/	1	5.772	1.055

* p = .05,

** p = .01

### 3.5 Predicting Behavioural Intention

There were in total two steps in predicting behavioural intention. Step (1) analysis: Attitude, Subjective Norm and Perceived Behavioural Control were entered as predictor variables in predicting behavioural intention. In step (2) analysis, apart from Attitude, Subjective Norm and Perceived Behavioural Control, Food Preference was entered to predict behavioural intention as predicting variable.

Table 5 presented the results of hierarchical regression analysis. In step (1) analysis, TPB model could predict about 48% of the variance of behavioural intention (R^2^ = .477, p < .001), where all constructs were significant predictors. Among all predictors, Attitude was dominant and could predict about 30% of the variances of behavioural intention, whereas Subjective Norm and Perceived Behavioural Control contributed about one-fourth of the prediction respectively.

**Table 5:** Predicting intention toward fruit and vegetable consumption by hierarchical regression analysis (betas)

	Step 1	Step 2
Attitude	.314 *	.143
Subjective Norm	.276 *	.285 *
Perceived Behavioural Control	.254 *	.171
Food Preference	/	.341 *
R^2^	.477 ***	.545 ***

* p < .05,

*** p < .001

In step (2) analysis, the result indicated that the variance of predicting behavioural intention increased by 7% after incorporating Food Preference as a predictor. The model was able to explain about 55% of the variance of behavioural intention (R^2^ = .545, p < .001). Food Preference could account for nearly one-third of the variance in predicting behavioural intention (R^2^ = .341, p < .05), however, the contributions of both Attitude and Perceived Behavioural Control dropped. In addition, Subjective Norm remained one of the significant predictors, which was responsible for about 29% of the variance of behavioural intention (R^2^ = .285, p < .05).

## 4. Discussion

According to the official statistics conducted by the Hong Kong Government (Centre for Health Protection), around 39% of population in Hong Kong were overweight in 2014. In this case study, the percentage of overweight students with mild ID was 58%, which was about 1.5 times of the figure of normal population in Hong Kong. This was an alarming figure and gave a snapshot of how serious the problem was in the population of students with mild ID.

Regarding F&V consumption in Hong Kong, the percentage of normal population consuming sufficient F&V was about 18.7% in 2014 (Centre for Health Protection), however, only 4% of students with mild ID had enough F&V consumption in this study. It meant more than 4 times difference between normal population and group of students with mild ID, which was in line with the findings of some studies (Draheim, Stanish, Williams & McCubbin, 2007; Heller, McCubbin, Drum, & Peterson, 2011). Peltzer and Pengpid reported that inadequate F&V consumption was one of the leading factors of overweight (Peltzer & Pengpid, 2012). This association outlined the picture of students with mild ID in Hong Kong, the lesser the F&V consumption the higher percentage of overweight was among them.

In the present study, TPB was employed to predict the intention of F&V consumption among students with mild ID. The result was promising as the model was able to account for about 48% of the variance of F&V consumption intention with attitude being the dominant predictor, following by subjective norm and perceived behavioural control. Attitude held the strongest correlation with behaviour intention which corresponded to about 30% of the variance of prediction. These findings were in line with other research on F&V consumption behaviour (Backman, Haddad, Lee, Johnston, & Hodgkin, 2002). When considering the presence of Food Preference as an additional construct, the predicting efficacy of TPB model for F&V consumption intention was slightly improved from 47.7% to 54.5%. Food preference was found to have the strongest association (r = .617, p = .01) with behavioural intention, whereas subjective norm remained more or less the same. For attitude and perceived behavioural control, their contributions were attenuated. From the works of Hartman, food preference was regarded as one of the important psychosocial determinants of F&V intake, this phenomenon was also noted in this study (Hartman, Wadsworth, Penny, Van Assema, & Page, 2013).

Several studies revealed that people in general population were less affected by social influence when predicting dietary behaviour (Dunn, 2008). It was most likely affected by either Attitude or Perceived Behavioural Control. In this study, Attitude was also found to be the major predictor of F&V consumption intention.

The correlations of Subjective Norm among parents, teachers and friends were similar, although parents were taking the leading role with slightly higher weighting as shown in Table 6. Guralnick explained that family members or parents were important in the influence on the development and behaviour of people with ID (Guralnick, Neville, Connor, & Hammond, 2003). It was believed that the social pressure from family members or parents was the strongest influence.

**Table 6:** Inter-correlation between subjective norm and important referents

	SN	Parents	Teachers	Friends
Subjective Norm (SN)	1	.764 **	.730 **	.699 **
Parents	/	1	.601 **	.558 **
Teachers	/	/	1	.648 **
Friends	/	/	/	1

** p = .01

Food Preference predicted about one-third of the variance of F&V consumption intention in this study, which was the dominant factor in the extended model. Since there was limited research available by applying the TPB model to examine relationship between Food Preference and dietary behavioural intention, present study could provide a new insight regarding to Food Preference. According to some research, it was believed that food preference facilitated people to consume more F&V due to the likeliness of tastes (Keim, Swanson, & Cann, 2001; Lautenschlager & Smith, 2007; Krolner et al., 2011). Studies of Hartman et al. concluded that the perceptions of taste decided people whether or not to eat F&V (Hartman, Wadsworth, Penny, Van Assema, & Page, 2013). In this study, similar result was found that Food Preference was one of the major predictors for F&V consumption intention among students with mild ID in Hong Kong.

## 5. Conclusion

The results of this study outlined the health behaviour of F&V consumption among students with mild ID in Hong Kong. The dietary pattern of minority group was unhealthy and far from the standard proposed by Department of Health. This ill-health behaviour leaded to various negative health consequences and put the students with mild ID at risk.

TPB was proved to be a useful behavioural model to predict F&V consumption intention. It successfully provided a framework for understanding the determinants of F&V consumption among students with mild ID in Hong Kong. Among the major constructs, Attitude, Subjective Norm and Perceived Behavioural Control, all three predictors had significant positive influences on dietary behavioural intention of F&V intake. This study gave solid evidence that Food Preference might be a strong predictor to explain F&V intake. It pointed out that interventions would not limit to Attitude, Subject Norm and Perceived Behavioural Control, but also by improvement of the Food Preferences, such as taste or sensory characteristics. It provided clues for health promotion practitioners and educators to rethink the current strategies on F&V consumption among students with mild ID in Hong Kong.

Several limitations in this study should be acknowledged. First, the sample size of this work was limited to one special school; it may not reflect the true picture of F&V consumption behaviour of students with mild ID in Hong Kong. Other than IQ, questions on mental health illnesses of participants were not permitted due to privacy policy. Regarding the reliability of TPB constructs, Perceived Behavioural Control was at the marginal acceptable line. Questions on this part should be revised in order to reduce the complexity of questions for students with mild ID. Last but not least, for better measurement, it will be better to take daily food log for a period of time in order to understand the accurate F&V consumption behaviour rather than food recall.
